# Validity evidence and psychometric evaluation of a socially accountable health index for health professions schools

**DOI:** 10.1007/s10459-023-10248-5

**Published:** 2023-06-22

**Authors:** Cassandra Barber, Cees van der Vleuten, Saad Chahine

**Affiliations:** 1 School of Health Professions Education (SHE), Faculty of Health, Medicine and Life Sciences, Maastricht University, Maastricht, The Netherlands; 2 Faculty of Education, Queen’s University, Kingston, ON Canada

**Keywords:** Social accountability, Population health, Psychometrics, Quantitative methods, Medical education

## Abstract

There is an expectation that health professions schools respond to priority societal health needs. This expectation is largely based on the underlying assumption that schools are aware of the priority needs in their communities. This paper demonstrates how open-access, pan-national health data can be used to create a reliable health index to assist schools in identifying societal needs and advance social accountability in health professions education. Using open-access data, a psychometric evaluation was conducted to examine the reliability and validity of the Canadian Health Indicators Framework (CHIF) conceptual model. A non-linear confirmatory factor analysis (CFA) on 67 health indicators, at the health-region level (n = 97) was used to assess the model fit of the hypothesized 10-factor model. Reliability analysis using McDonald’s Omega were conducted, followed by Pearson’s correlation coefficients. Findings from the non-linear CFA rejected the original conceptual model structure of the CHIF. Exploratory post hoc analyses were conducted using modification indices and parameter constraints to improve model fit. A final 5-factor multidimensional model demonstrated superior fit, reducing the number of indicators from 67 to 32. The 5-factors included: Health Conditions (8-indicators); Health Functions (6-indicators); Deaths (5-indicators); Non-Medical Health Determinants (7-indicators); and Community & Health System Characteristics (6-indicators). All factor loadings were statistically significant (*p* < 0.001) and demonstrated excellent internal consistency ($\upomega$ >0.95). Many schools struggle to identify and measure socially accountable outcomes. The process highlighted in this paper and the indices developed serve as starting points to allow schools to leverage open-access data as an initial step in identifying societal needs.

## Introduction

Health professions education aims to produce competent graduates equipped to meet societal needs. This goal represents one of the core principles of social accountability in medical education, which emphasizes the need for schools to direct their education, research, and service activities towards priority health needs of the communities they serve (Boelen & Heck, 1995; Global Consensus for Social Accountability of Medical Schools, 2010). However, there remains a misalignment between health professions education and societal needs (Ross and Cameron, 2021). While many schools have explicit institutional mandates to serve a specific geographic area or region (Barber et al., 2022), schools often remain unaware of the local health needs in their communities (Global Consensus and for Social Accountability of Medical Schools, 2010). One approach to address this gap is for schools to leverage open access, secondary population health data to better identify priority health needs. Despite repeated calls to utilize publicly available data to improve medical training (Triola et al., 2018; Chahine et al., 2018; Dauphinee, 2012), this data has yet to be fully utilized to identify societal needs. This paper demonstrates how open access pan-national population health data can be used to better identify relevant health needs and advance the social accountability mandate of health professions education.

### Social accountability in medical education

Social accountability in health professions education is the obligation of medical schools to actively address the priority health needs of to their local communities (Boelen & Heck, 1995; Global Consensus and for Social Accountability of Medical Schools, 2010). This includes ensuring education, research, and service activities are aligned with societal needs. Social accountability represents a measurable activity (Global Consensus and for Social Accountability of Medical Schools, 2010), rooted in the identification of priority health needs and evaluated based on how well those needs are met (Dauphinee, 2012; Barber et al., 2020; Ventres et al., 2018; Boelen et al., 2019; Palsdottir et al., 2008; Larkins et al., 2013; Ross et al., 2014; Strasser et al., 2015; Preston et al., 2016). One strategy for schools to better identify priority health needs is to leverage open-access, secondary population health data.

### Population health data and education

Pan-national population health data are collected iteratively by governments or non-profit agencies in most countries worldwide for research, public policy, evaluation, and accountability purposes. This data is used extensively in public health, epidemiology, as well as social, health and clinical sciences. However, despite repeated calls to better utilize publicly available data to improve medical training (Triola et al., 2018; Chahine et al., 2018; Dauphinee, 2012), this data has yet to be leveraged to better inform educational, research and service activities.

Health indicators, derived from population health data, are often represented as summary statistics or proxy measures of health and factors that influence health (Idler & Benyamini 1997; Ashraf et al., 2019). They are often used to evaluate population health outcomes and health systems performances through advocacy, accountability, quality improvement, and research (Etches et al., 2006; Murray & Frenk 2000). Health indicators provide insights into health risks, patterns, and trends and determine the extent to which performance expectations are met (Declich & Carter, 1994). These indicators are often used for accountability purposes by governments, health professionals, voluntary agencies, and the public. Additionally, health indicators are also used to improve public health education and professional training (Murray et al., 2002; Porter, 1993). Health indicator frameworks capture relevant health outcomes, often comprised of numerous health and non-health related measures, to assess and monitor population health outcomes, inequities, and health care utilization (Ashraf et al., 2019; Etches et al., 2006; Kindig & Stoddart, 2003; Braithwaite et al., 2017). However, many health indicator frameworks lack validity evidence as they are often developed using conceptual models (Ashraf et al., 2019; Etches et al. 2006). Despite their usefulness in explaining causal connections and interrelationships across specific domains, these frameworks must be empirical evaluated to ensure reliability and determine their effectiveness in serving their intended purposes (Etches et al., 2006; Krieger, 2001).

Our review of the health professions education literature provides some key examples and methods of how population health data can be used to set educational priorities (MacDonald et al., 1989), inform curricular content (Arthur & Baumann 1996), and evaluate institutional practices (Coutinho et al., 2017). For instance, MacDonald et al., (1989) utilized secondary population health data to inform curricular content and establish educational priorities across the health professions training continuum. While this article was published more than 30 years ago, the authors identified prevalent health conditions in a population or geographic area to better inform curricular planning and set educational priorities. Their goal was to better equip medical graduates to address priority health needs of the community they serve. Similarly, Arthur & Baumann (1996) described a planning framework to identify essential curricular context using a mixed methods approach. The authors utilized secondary population health data to identify community health needs relevant to nursing education. This data was triangulated using an expert panel and review of the literature to help inform core curricular content surrounding priority health issues. Lastly, Coutinho et al., (2017) examined the relationship between primary care medical graduates and indicators of population need using demographic data obtained from the United States Census Bureau. Findings from this study suggest little correlation between primary care residency training and population need. Moreover, the strategic initiative of expanding primary care residency training was not correlated to state needs in terms of the number of primary care physicians per population (Coutinho et al., 2017).

This paper adds to the literature by leveraging open-access, pan-national population health data and validates its viability to assist schools identify relevant health needs for social accountability purposes. This work is imperative in advancing the social accountability agenda of health professions education and can be used to identify regional health needs, inform educational priorities, and perhaps serve as an initial step towards monitoring educational outcomes on population health.

## Methods

The goal of this study was to put forward an evidenced-based model that can be used by others to support social accountability. In this paper, we used open-source pan-national data from Statistics Canada’s Canadian Community Health Survey (CCHS) Public Use Microdata File (PUMF) (Statistics Canada, 2021) and online mortality and vital statistics (Statistics Canada, 2018) to examine the factor structure and reliability of a national conceptual health indicator model in Canada. Using an iterative approach, a non-linear factor analysis was used to validate the viability of the Canadian Health Indicator Framework (CHIF) (Canadian Institute and for Health Information, 2013).

### Study setting

Canada was the first country to adopt a national social accountability mandate for medical education globally (Health Canada, 2001). The Canadian healthcare system is publicly funded and provides universal coverage for medically necessary hospital and physician services to all Canadian citizens and permanent residents. The system is primarily funded through taxpayers and managed by individual provinces or territories (Government of Canada, 2023). Canada also provides open access to high-quality and easily accessible pan-national data on the economy, society, and environment (Statistics Canada, 2020a). Currently, 187 countries worldwide have national statistical systems that collect, process, and disseminate official statistics on behalf of their respective national governments (Open Data Watch, 2020). These systems aim to provide relevant, comprehensive, accurate, and objective statistical information on a country’s society, economy, and environment (Statistics Canada 2016a).

Canada is widely recognized for having some of the most comprehensive health data in the world (Lucyk et al. 2015). However, unlike other countries, Canada has yet to widely adapted a reliable national health indicator framework (Office of the Auditor General of Canada, 2008).

### Organizational framework

The CHIF (Canadian Institute and for Health Information, 2013) was selected as the organizational framework for grouping variables available from Statistics Canada’s 2017–18 CCHS PUMF (Statistics Canada, 2021) and online mortality and vital statistics.

The CHIF is a conceptual model developed by Statistics Canada and Canadian Institute for Health Information (CIHI) through national consensus with provincial and regional health authorities (Canadian Institute and for Health Information, 2013). Statistics Canada is Canada’s national statistical agency responsible for collecting statistical data on the country’s population, economy, society, and culture (Statistics Canada, 2020b). CIHI is an independent, not-for-profit organization that works closely with Statistics Canada and provincial and territorial governments to collect and share data on Canada’s health system and population health (Canadian Institute and for Health Information, 2021).

This framework provides reliable and comparable data on the health of Canadians, health care systems, and health determinants (Canadian Institute and for Health Information, 2021). It consists of over 80 indicators, measured across 4 domains and several factors, including health status (4 factors), non-medical determinants of health (3 factors), health system performance (1 factor), and community health system characteristics (2 factors) (depicted in Table 1) (Canadian Institute and for Health Information, 2021). A more detailed description of the CHIF is provided on Statistics Canada and CIHI’s website (Canadian Institute and for Health Information, 2013). These indicators serve as both measures of health and factors which influence health, used to inform health policy and manage the health care system (Statistics Canada, 2021). The CHIF has been widely used in guiding previous health indicator development (Statistics Canada, 2015a). However, it has not been empirically validated.

**Table 1:** Canadian Health Indicator Framework (CHIF) conceptual model used in the selection of variables and development of the non-linear Confirmatory Factor Analysis (CFA) social health index

Constructs and sub-components	Summary of indicators
Health Status	
Well-Being	3 Broad measures assessing the physical, mental, and social well-being of individuals
Health Conditions	Inclusive of 17 items assessing individual attributes of health status which may lead to distress, interferences with daily activities, or contact with health services. These items are inclusive of disease (acute or chronic); injury or trauma; or health related status (e.g., birth-related indicators, aging, stress, or genetic predisposition)
Health Functions	5 Items assessing levels of human function associated with the consequence of disease, disorder, injury and other health conditions (e.g., body function/structure (impairments), activity limitations, restrictions in participations)
Deaths	11 Items assessing a range of age- and condition specific mortality rates as well as derived indicators
Non-Medical Determinants of Health	
Healthy Behaviours	8 Items assessing aspects of personal behaviour and risk factors that epidemiological studies have shown to influence health status
Living and Working Conditions	13 Indicators related to the socio-economic characteristics of working conditions of the population that epidemiological studies have shown to be related to health
Personal Resources	2 Items assessing prevalence of factors (social support) that epidemiological studies have shown to be related to health
Environmental factors	5 Environmental items with the potential to influence human health
Health System Performance	
Acceptability	1 Item measuring patient satisfaction with the care/services provided
Accessibility	Six items measuring the ability of patients to obtain health care/services, based on respective needs
Appropriateness	2 Items assessing the care/services provided is relevant to the clients’/patients’ needs and based on established standards
Continuity	1 Item assessing the ability to provide uninterrupted, coordinated care/services across programs, practitioners, organizations, and levels of care/services, over time
Effectiveness	10 Items assessing whether care/service intervention or action achieves the desired results
Safety	1 Item assessing potential risks of an intervention, or the environment are avoided or minimized
Community and Health System Characteristics	
Community	10 Items assessing community characteristics
Health System	13 Items assessing health system characteristics

The importance of developing a population health profile has been well-established in the literature (Boelen & Heck, 1995; Global Consensus and for Social Accountability of Medical Schools, 2010; Ventres et al., 2018). From a social accountability perspective, the local community serves as the main stakeholder of all health professions schools, and it is essential for schools to identify and respond to the priority health needs in the communities they serve (Boelen & Heck, 1995). This includes identifying and understanding the cultural context, social determinants of health, and health disparities in the communities they are expected to serve.

The CHIF serves as a comprehensive set of health indicators that are specifically designed to measure and monitor the health of Canadians. This framework may be used as a valuable tool for schools to their advance their social accountability mandate by identifying relevant population health needs in their respective geographic areas or region.

## Data

This study utilized two open-source data sources were, the CCHS PUMF and publicly available mortality and vital statistics data obtained online from Statistics Canada website.

The CCHS is a voluntary, cross-sectional nationally representative survey offered in both English and French and is distributed annual to individuals >12 years of age living in Canada (Statistics Canada, 2021). Excluded from the sampling frame are individuals living on Indigenous reserves or other settlements, full-time members of the Canadian Forces, institutionalized populations, children aged 12–17 living in foster care, and those living in remote health regions in Quebec (Statistics Canada, 2021). The survey employs a stratified multistage sampling strategy to provide reliable estimates at the health region level every two years (Statistics Canada, 2021).

The CCHS is comprised of two years of data and includes responses surveyed over the reference period. The CCHS cycle is comprised of common content (asked of all respondents), optional content (selected by each province/territory), and rapid response content (Statistics Canada, 2021). The common content collected during the first year of the survey cycle consists of questions asked of all respondents. The optional content, collected from a smaller sample during the second year of the survey cycle, comprises of questions selected by each province/territory on specific health topics (Statistics Canada, 2021).

The CCHS PUMF is an open access dataset representing 3% of the Canadian population, inclusive of approximately 1,050 variables related to Canadians' health-status, health care utilization, and health determinants, including socio-demographic data, health conditions and diseases, lifestyle, social conditions, as well as mental health and well-being. A more detailed description of the CCHS PUMF survey design, sampling methodology, and validation has been described elsewhere (Statistics Canada, 2021).

To ensure comprehensive representation of all factors associated with the CHIF, publicly available mortality, vital statistics, and community indicators data were obtained online from Statistics Canada’s website (Canadian Institute and for Health Information, 2013; Statistics Canada, 2016b).

Ethics approval was obtained from Maastricht University’s Ethics Review Committee Health, Medicine and Life Sciences (FHML*-*REC).

### Analysis

#### Level of analysis

Due to missing data observed at the individual level due to the CCHS sampling design and data disclosures controls, the level of analysis was aggregated to the health region level (n = 97). The CCHS employs a stratified multistage sampling cycle and imposes several data disclosure controls to protect respondent anonymity and confidentiality. These controls include the use of subsampling and data suppression techniques, such as the removal of sensitive variables (e.g., outliers) or indirect identifiers (i.e., socio-demographic characteristics, geographic metrics), to minimize the risk of disclosing personal information due to small population sizes. These methods minimize the potential for identifying individual respondents while preserving the analytical value of the data (Statistics Canada, 2021).

To overcome missing data issues observed at the individual level, place-level data aggregation was imposed at the health region level. Health regions are administrative areas defined by provincial ministries of health responsible for delivering public health care services (Statistics Canada, 2018). Aggregating the CCHS PUMF at the health region level yielded a total analytical sample of 97 health regions, which are listed in ‘Appendix I’.

#### Measures

The selection of health indicators was guided by the CHIF conceptual model and based on data availability from the 2017–18 CCHS PUMF, and mortality and vital statistics, and community indicators obtained online from Statistics Canada website (Canadian Institute and for Health Information, 2013; Statistics Canada, 2016b). A total of 67 variables were identified and selected to measure the CHIF conceptual model across four domains and several factors and indicators: (1) health status (4 factors, 40 indicators), (2) non-medical determinants of health (3 factors, 17 indicators), (3) health system performance (2 factors, 2 indicators), and (4) community health system characteristics (2 factors, 8 indicators) (shown in Table 2).

**Table 2:** Mean, SDs, skewness, kurtosis, and range of possible scores for the variables included in the non-linear confirmatory factor analysis (CFA), Canadian Community Health Survey (CCHS) Public Use Mircodata File (PUMF), 2017–18

Domains, factors, and indicators	No. (%) out of a possible 97 cases	Mean (SD)	Skewness (SE)	Kurtosis (SE)	Range of Possible Scores
Health Status					
Well-Being					
Perceived Health	97 (100.00)	1.03 (0.42)	1.84 (0.25)	4.15 (0.49)	0.51–2.79
Perceived Mental Health	97 (100.00)	1.03 (0.53)	2.42 (0.25)	7.62 (0.49)	0.51–3.64
Perceived Life Stress	97 (100.00)	1.03 (0.59)	2.18 (0.25)	5.78 (0.49)	0.40–3.39
Health Conditions					
Adult BMI	97 (100.00)	1.03 (0.48)	1.99 (0.25)	5.03 (0.49)	0.48–3.05
Youth BMI	97 (100.00)	1.03 (0.54)	1.78 (0.25)	4.15 (0.49)	0.31–3.10
Arthritis	97 (100.00)	1.03 (0.42)	1.55 (0.25)	3.78 (0.49)	0.33–2.78
Diabetes	97 (100.00)	1.03 (0.45)	1.29 (0.25)	2.33 (0.49)	0.28–2.80
Asthma	97 (100.00)	1.03 (0.52)	2.03 (0.25)	5.60 (0.49)	0.39–3.35
High Blood Pressure	97 (100.00)	1.03 (0.42)	1.52 (0.25)	3.12 (0.49)	0.41–2.69
Chronic Obstructive Pulmonary Disease (COPD)	97 (100.00)	1.03 (0.46)	1.14 (0.25)	1.18 (0.49)	0.31–2.51
Pain or Discomfort that Prevents Activities	97 (100.00)	1.03 (1.17)	3.18 (0.25)	13.28 (0.49)	0.07–7.89
Pain or Discomfort by Severity	97 (100.00)	1.03 (1.13)	2.94 (0.25)	11.00 (0.49)	0.07–7.23
Mood Disorders	97 (100.00)	1.03 (0.52)	2.45 (0.25)	8.20 (0.485)	0.45–3.60
Low Birth Weight	96 (98.90)	1.04 (1.45)	3.37 (0.25)	13.09 (0.49)	0.12–9.14
High Birth Weight	96 (98.90)	1.03 (0.91)	2.39 (0.25)	7.15 (0.49)	0.06–5.38
Small for Gestational Age	96 (98.90)	1.04 (1.57)	3.47 (0.25)	13.74 (0.49)	0.11–9.91
Large for Gestational Age	96 (98.90)	1.04 (1.00)	2.60 (0.25)	8.21 (0.49)	0.13–6.04
Pre-Term Births	96 (98.90)	1.04 (1.32)	3.17 (0.25)	11.64 (0.49)	0.11–8.17
Cancer Incidence (Lifetime)	97 (100.00)	1.03 (0.46)	1.53 (0.25)	4.22 (0.49)	0.28–3.06
Injury Required Hospitalization	97 (100.00)	1.03 (0.48)	2.21 (0.25)	6.71 (0.49)	0.27–3.19
Injury Requiring Medical Attention (24 hours)	97 (100.00)	1.03 (0.55)	2.90 (0.25)	11.83 (0.49)	0.42–3.93
Suffers from Effects of a Stroke	97 (100.00)	1.03 (0.48)	1.42 (0.25)	2.91 (0.49)	0.26–2.89
Health Functions					
Difficulty Seeing	97 (100.00)	1.03 (0.49)	2.34 (0.25)	6.88 (0.49)	0.50–3.23
Difficulty Hearing	97 (100.00)	1.03 (0.44)	2.12 (0.25)	5.78 (0.49)	0.50–2.84
Difficulty Walking/Climbing Steps	97 (100.00)	1.03 (0.43)	1.74 (0.25)	4.40 (0.49)	0.43–2.76
Difficulty Remembering/Concentrating	97 (100.00)	1.03 (0.49)	2.08 (0.25)	5.62 (0.49)	0.46–3.16
Difficulty Self-Care	97 (100.00)	1.03 (0.43)	1.94 (0.25)	5.18 (0.49)	0.46–2.90
Difficulty Communicating	97 (100.00)	1.03 (0.48)	1.67 (0.25)	3.89 (0.49)	0.38–3.05
Participation and Activity Limitation	97 (100.00)	1.03 (1.14)	3.18 (0.25)	13.41 (0.49)	0.08–7.68
Deaths					
Infant Mortality	87 (89.70)	1.14 (1.39)	3.53 (0.26)	15.75 (0.51)	0.29–9.41
Perinatal Mortality	92 (94.80)	1.09 (1.42)	3.63 (0.25)	16.51 (0.50)	0.23–9.72
Total Mortality	97 (100.00)	1.03 (1.03)	3.34 (0.25)	14.76 (0.49)	0.06–6.69
All Diseases of the Circulatory System Deaths	97 (100.00)	1.04 (1.00)	3.05 (0.25)	12.57 (0.49)	0.03–6.25
All Malignant Neoplasms (Cancer) Deaths	97 (100.00)	1.03 (1.04)	3.18 (0.25)	13.18 (0.49)	0.05–6.46
All Diseases of the Respiratory System Deaths	97 (100.00)	1.03 (0.98)	3.20 (0.25)	13.48 (0.49)	0.09–6.20
Suicide	97 (100.00)	1.03 (1.03)	2.67 (0.25)	8.57 (0.49)	0.13–5.92
Unintentional Injury Deaths	97 (100.00)	1.03 (0.89)	3.39 (0.25)	17.59 (0.49)	0.12–6.72
Premature Mortality	95 (97.90)	1.05 (0.94)	2.73 (0.25)	9.74 (0.49)	0.13–5.98
Potential Years of Life Lost–for Total Mortality	95 (97.90)	1.05 (1.00)	2.89 (0.25)	10.79 (0.49)	0.18–6.51
Non-Medical Determinants of Health					
Healthy Behaviours					
Smoking	97 (100.00)	1.03 (0.49)	1.55 (0.25)	2.70 (0.49)	0.41–2.88
Heavy Drinking	97 (100.00)	1.03 (0.54)	2.10 (0.25)	5.37 (0.49)	0.44–3.37
Adult Physical Activity Indictor based on Canadian Physical Activity Guidelines	97 (100.00)	1.03 (0.50)	1.40 (0.25)	2.10 (0.49)	0.32–2.76
Adult (18+) Self- Reported Physical Activity, 150 min/week	97 (100.00)	1.03 (0.50)	1.40 (0.25)	2.10 (0.49)	0.32–2.76
Youth Physical Activity Indictor based on Canadian Physical Activity Guidelines	97 (100.00)	1.03 (0.52)	1.59 (0.25)	3.31 (0.49)	0.39–3.08
Youth (12–17 yrs old) Self-Reported Physical Activity, Average 60 min/day	97 (100.00)	1.03 (0.53)	1.37 (0.25)	2.27 (0.49)	0.33–2.92
Breastfeeding Practices	97 (100.00)	1.03 (0.72)	1.90 (0.25)	4.12 (0.49)	0.24–3.99
Living and working conditions					
Education (High School or Less)	97 (100.00)	1.03 (0.43)	1.26 (0.25)	1.35 (0.49)	0.46–2.35
Unemployment Rates	97 (100.00)	8.8 (3.93)	2.53 (0.25)	7.66 (0.49)	4.50–27.64
Long-Term Unemployment Rate	97 (100.00)	1.03 (1.34)	3.68 (0.25)	15.74 (0.49)	0.15–8.77
Low-Income Rate	97 (100.00)	1.03 (1.89)	4.97 (0.25)	29.54 (0.49)	0.00–14.16
Median Share of Income	97 (100.00)	22.08 (1.45)	− 1.62 (0.25)	4.06 (0.49)	16.40–24.30
Government Transfer Income	97 (100.00)	14.07 (4.22)	0.47 (0.25)	0.77 (0.49)	5.50–28.30
Housing Affordability	97 (100.00)	1.03 (1.61)	4.59 (0.25)	26.91 (0.49)	0.02–12.21
Household Food Insecurity	97 (100.00)	1.03 (0.61)	2.41 (0.25)	6.53 (0.49)	0.32–3.70
Personal Resources					
Sense of Community Belonging	97 (100.00)	1.03 (0.70)	1.95 (0.25)	4.04 (0.49)	0.38–3.64
Life Satisfaction	97 (100.00)	1.03 (0.56)	2.21 (0.25)	6.11 (0.49)	0.37–3.63
Health System Performance					
Accessibility					
Influenza Immunization	97 (100.00)	1.03 (0.49)	2.13 (0.25)	6.054 (0.485)	0.52–3.18
Regular Medical Doctor	97 (100.00)	1.03 (0.50)	1.90 (0.25)	5.715 (0.485)	0.12–3.37
Community and Health System Characteristics					
Community					
Rural Population	96 (98.90)	1.05 (0.68)	1.07 (0.25)	1.112 (0.488)	0.01–3.08
Indigenous Population	97 (100.00)	1.03 (1.27)	3.76 (0.25)	17.639 (0.485)	0.00–8.79
Immigrant Population	97 (100.00)	12.83 (12.17)	1.80 (0.25)	3.21 (0.485)	0.00–60.2
Internal Migrant Mobility	97 (100.00)	1.03 (0.97)	1.81 (0.25)	3.165 (0.485)	0.12–4.64
Lone-Parent Families	97 (100.00)	1.03 (0.53)	1.67 (0.25)	3.198 (0.485)	0.37–3.04
Visible Minority Populations	97 (100.00)	1.03 (2.55)	4.38 (0.25)	23.237 (0.485)	0.01–18.06
Health System					
Contact with a Medical Doctor/Health Care Professional (Last 12 Monthsths)	97 (100.00)	1.03 (1.23)	3.03 (0.25)	11.796 (0.485)	0.05–8.02
Contact with Dental Professionals (Last 12 Months)	97 (100.00)	1.03 (0.51)	2.06 (0.25)	5.416 (0.485)	0.48–3.29

Nominal and ordinal scale indicators were recoded dichotomously. For instance, non-favourable health outcomes such as fair or poor perceived health, presence of disease (e.g., arthritis, cancer, diabetes, high blood pressure, etc.), and personal behaviours and risk factors (e.g., under/overweight, or obese body mass index (BMI), smoking, heavy drinking, etc.) were coded as ‘1’. On the other hand, favourable health outcomes such as good, very good, or excellent perceived health, normal BMI, absence of disease (e.g., no cancer in lifetime, normal blood pressure), and positive personal behaviours (e.g., non-smoker or non-drinker, etc.) were coded as ‘0’. These indicators were aggregated to the health region level and calculated as proportions derived from discrete counts at the aggregated health region. Ratio-scale variables such as income (i.e., low-income rates, medium share of income, government transfer income) and employment rates (unemployment rate, long-term unemployment rate) were not dichotomized to preserve their continuous scale and were aggregated to the health region level. The analytical dataset comprised of compositional data derived from discrete count-based proportions or percentages aggregated to the health region level (Aitchison, 1982).

#### Analytical approach

To assess the factor structure of the CHIF at the health region level, a non-linear confirmatory factor analysis (CFA) was used due to the non-normality of the data (McDonald, 1967; Bauer & Hussong, 2009).

Validity frameworks often consist of four components, including content validity, response process validity, internal structure validity, and criterion validity (Smirnova et al., 2022; Cook et al., 2015). The rigorous design and development process of the CHIF involved three validity components: content validity, response process validity, and criterion validity. These validity components were established through a comprehensive review of existing literature and expert consultation, the use of clear operational definitions and standardized data collection methods, and comparison with other established measures of health status (Canadian Institute and for Health Information, 2013). This paper specifically assesses the internal structure validity of the CHIF using CFA. Factor analyses are often utilized to provide construct validity evidence (Henson & Roberts, 2006; American Education Research Association et al., 1999; Thompson & Daniel, 1996) to evaluate the underlying structure of the observed measures by examining inter-item correlation.

Using an iterative process, maximum likelihood with robust standard errors (MLR) and accelerated expectation maximization (EMA) estimators were used to estimate the factors. The expectation maximization (EM) algorithm (Byrne, 2005) was used to optimize the complete data loglikelihood, while EMA, an accelerated EM procedure, utilized Quasi-Newton and Fisher Scoring optimization (Schreiber et al., 2006). To improve model fit through modifications indices and identify potential misspecified parameters, post hoc model fit was conducted in an exploratory manner (Dempster et al., 1977). This approach aimed to create a multi-dimensional respecified model while ensuring that the hypothesized model fit well with the observed data and aligned theory and epistemology (Muthén & Muthén, 2007). CFA analyses were conducted in Mplus (Version 8.7, Muthén & Muthén, Los Angeles, CA).

#### Model specification

The CHIF conceptual model was used to initially specify the factor structure of the model. Criteria for retaining items in the model included a statistically significant path coefficient (*p* < 0.05) between the item and its predicted subscales on the CHIF. Post-hoc modification indices were used to modify the model for improved model fit indices. To set a metric for each factor, unit loading identification constraints were imposed by fixing the unstandardized coefficient of one item per latent variable equal to one (Kline, 2015).

The respecification process included examining modification indices, residuals, parameter estimates, and explained variance. Based on these sources of the model information, the Well-Being factor and several indicators within the five remaining hypothesized factors were deleted due to weak relationships and excessive redundancy of items (Wang & Staver, 2001). The use of modification indices resulted in the identification of additional statistically significant paths, leading to a better model fit. All factor loadings were statistically significant (*p* < 0.001), and residuals remained close to zero.

Several conditions needed to be satisfied for an item to be retained in the generated model. The path coefficient between an item and its predicted subscale on the CHIF needed to be statistically significant (*p* < 0.05). Post-hoc modification indices generated from the structural parameters were used to modify the model to achieve better model fit indices. To set a metric for each factor, unit loading identification constraints were imposed (Kline, 2015); the unstandardized coefficient of one item per latent variable was fixed equal to ‘1’.

Respecification of the structural model included the examination of the following: (1) modification indices, (2) residuals, (3) parameter estimates, and (4) explained variance. Taken together, sources of model information suggested the deletion of the Well-Being factor as well as several indicators within the five remaining hypothesized factors. Item deletion was deemed appropriate due to weak relationships and evidence of excessive redundancy of items (Hoyle, 1995). Additionally, modification indices generated from the structural parameters were used to identify additional statistically significant paths, resulting in a better model fit. All factor loadings were statistically significant (*p* < 0.001), and residuals remained close to zero.

#### Model fit

The quality of the model was assessed by examining several fit indices, including Chi-square (χ^2^), Comparative Fit Index (CFI), Tucker-Lewis Index (TLI), Root Mean Square Error of Approximation (RMSEA), and Standardized Root-Mean-Square Residuals (SRME). Model fit was evaluated using a combination of these indices (Hoyle, 1995; Thompson, 2004). The following thresholds were selected based on previous literature: CFI and TLI values ≥ 0.95 were considered favourable and indicative of good model fit (Hu & Bentler 1998), RMSEA values between 0.05 and 0.08 indicated reasonable error of approximation (Browne & Cudeck, 1992), and SRMR values ≤ 0.08 were considered reasonable (Browne & Cudeck, 1992).

#### Reliability

Internal consistency of scales resulting from the final CFA model was assessed using McDonalds Omega $\upomega$ coefficient. The coefficient was obtained in JAMOVI (Version 1.2; The jamovi project, Sydney, Australia). McDonald’s Omega coefficient was preferred over Cronbach’s Alpha as it has been suggested to have superior psychometric properties and provide more accurate estimates of a scale’s internal structure (Crutzen & Peters, 2017; Peters, 2014; Revelle & Zinbarg, 2009).

## Results

In total, 67 indicators aggregated to the health region level (n = 97) from the 2017–18 CCHS PUMF and online mortality and vital and community indicators were analyzed using non-linear CFA. Table 3 provides the mean, standard deviation, distribution (skewness & kurtosis), and range of possible scores for the variables included in analysis. Overall, the number of health regions per indicator remained relatively stable. However, the range of possible scores, means, and standard deviation for each indicator varied. The skewness and kurtosis measures confirm non-normality of all indicators, except for the Government Share Income indicator.

**Table 3:** Model fit indices for social health indices

Model		Fit index					$\upomega$	Mean (SD)
		χ^2^	TLI	CFI	RMSEA	SRME		
1.	Health Conditions	16.617*	0.993	0.997	0.073	0.015	0.964	7.22 (3.57)
2.	Health Functions	9.478*	0.996	0.998	0.044	0.009	0.967	6.19 (3.09)
3.	Deaths	7.590*	0.994	0.997	0.073	0.007	0.984	5.15 (5.24)
4.	Non-Medical Health Determinants	19.416*	0.988	0.993	0.080	0.012	0.979	7.22 (3.50)
5.	Community & Health System Characteristics	8.733*	0.994	0.997	0.051	0.011	0.945	6.19 (3.08)

*Χ*^2^ Chi-square; *TLI*  Tucker–Lewis index; *CFI* Comparative fit index; *RMSEA* Root mean square error of approximation; *SRME*  Standardized root mean error; $\omega$ McDonald’s Omga; and *SD* Standard deviation

Recommended cut-offs: *χ*^2^ (p < 0.05); TLI & CFI (> 0.95); RMSEA (< 0.05–0.08); SRME (< 0.08)

### Social health index

**Fig. 1:**
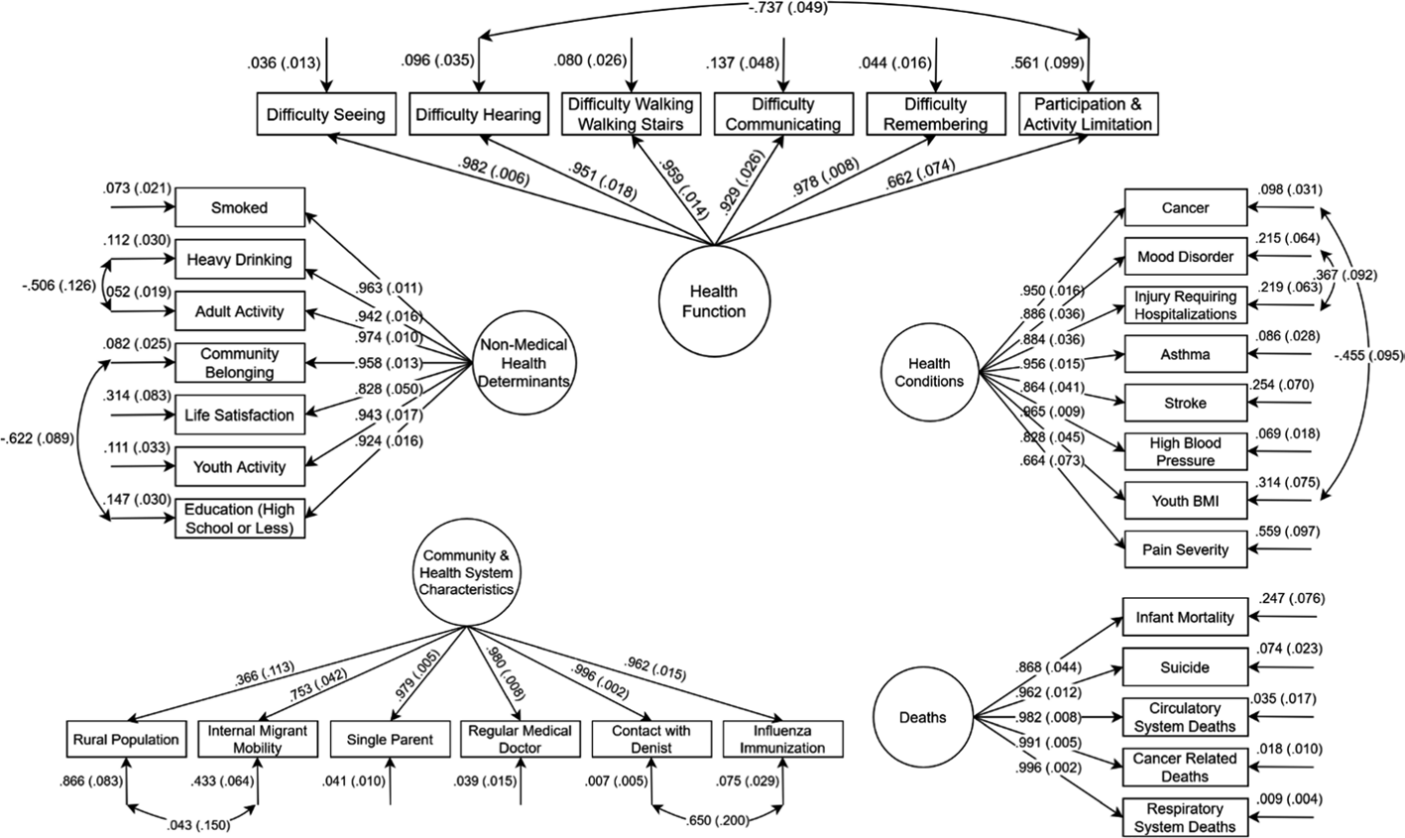
Final model with standardized loadings for 32 health indicators aggregated to the health region level from the 2017–18 CCHS PUMF. Observed variables are represented as rectangles, circles represent the unobserved variables, and the arrows going to the rectangles represent the measurement error associated with each observed variable. The arrows between unobserved and observed variable represents a regression path and the standardised regression weight. The double-headed arrows represent the correlation between two unobserved variables (factor covariances) in the model

The initial model involved 67 measured indicators and 10 hypothesized factors (shown in Table 2). However, the initial 10-factor model was rejected due to poor model fit. Post hoc analyses were conducted in an exploratory manner to identify which parameters in the model were misspecified (Aitchison, 1982). Using an iterative process, modification indices and parameter constraints were imposed to improve model fit. The final 5-factor CFA (depicted in Fig. 1) included: (1) Health Conditions (8 indicators), (2) Health Functions (6 indicators), (3) Deaths (5 indicators), (4) Non-Medical Health Determinants (7 indicators), and (5) Community & Health System Characteristics (6 indicators).

Overall, 35 indicators were removed, resulting in the reduction of the number of indicators in the initial model from 67 to 32. Additionally, seven correlated error terms were allowed between two indicators on four of the five factors: Health Conditions, Health Functions, Non-Medical Health Determinants, and Community & Health System Characteristics. The 5-factor model demonstrated good model fit according to the recommended criteria (Kline, 2015) (shown in Table 4).

**Table 4:** Correlation matrix of the social health indices

		1	2	3	4
1	Health Conditions				
2	Health Functions	0.986*			
3	Deaths	0.819*	0.819*		
4	Non-Medical Health Determinants	0.956*	0.943*	0.733*	
5	Community & Health System Characteristics	0.955*	0.940*	0.788*	0.945*

*p ≤ 0.001

Internal consistency reliability was conducted for the five scales at the health region level, based on the items retained in the final model, and assessed using McDonald’s ω coefficient (Table 4). The coefficients for all subscales were excellent, ranging from 0.945 to 0.984 (Lucke, 2005).

Pearson’s correlation coefficients were used to investigate the inter-relationships between the CFA factors. As shown in Table 4, all correlation coefficients were significant and positively correlated with one another. Based on the magnitude of the coefficients (ranging from strong to very strong), the strength of the association was highest for Health Function and Health Conditions (0.986), and lowest for Non-Medical Health Determinants and Deaths (0.733).

## Discussion

This study developed and evaluated a multi-dimensional health index to be used by health professions programs for social accountability purposes. Utilizing open access, pan-national health data, this paper assessed the psychometric properties and internal factor structure of an existing national conceptual health indicator framework in Canada. This study represents, to our knowledge, the first examination of the underlying factor structure and reliability assessment of the CHIF at the health region level. This effort extends previous research that examined the correlations between CHIF health and healthcare performance indicators at the provincial and territorial level (Arah & Westert, 2005).

Results from our non-linear CFA rejected the original 10-factor conceptual model structure of the CHIF. Exploratory post hoc analyses resulted in a 5-factor multi-dimensional model, demonstrating excellent model fit on various fit indices. Our findings, generally corroborate the structural validity of the CHIF. However, several modifications were imposed to improve model fit, reducing the number of constructs and indicators in the final model from 67 to 32, creating a more parsimonious set of indicators. Additionally, outcomes from our analysis did not psychometrically support the inclusion of the well-being construct at health region level due to poor model fit. However, this finding does not suggest that well-being is not an important health indicator. These reductions improved the feasibility and utility of the indices. The reliability of each subscale supported by McDonald’s $\upomega$ coefficient exceeded the recommended standards of > 0.80 (Lucke, 2005), indicating high internal consistency.

The findings from our 5-factor non-linear CFA demonstrated a multidimensional model of health, supportive of the multifaceted nature of the concept of health. The concept of health is both influenced and produced by biological and social factors, culture referents, as well as social interactions and networks (WHO, 1986; Olafsdottir, 2013; Conrad & Baker, 2010). These 5-factors may be used as parcels in examining health at the construct level (Matsunaga, 2008). These findings are consistent with the public health literature which favour multi-dimensional models of health, over a single health composite score (Braithwaite et al., 2017; Smith Papanicolas, 2013). The use of a single health composite measure was initially thought to provide a holistic overview of health and the healthcare system (Braithwaite et al., 2017; Smith Papanicolas, 2013). However, it has been found to be challenging to interpret and fails to account for heterogenous system differences (Braithwaite et al., 2017; Smith Papanicolas, 2013).

The process highlighted in this paper and the indices developed serve as starting points to allow schools to leverage open access population health data to better identify relevant priority health needs. This initial step in identifying community needs is imperative to advancing the social accountability agenda of health professions schools and may begin to close the gap between education and society. There are a number of ways in which this study might be used in the selection and teaching of medical students. From a programmatic standpoint these indices may be used by schools to better identify societal health needs, create community profiles, inform educational priorities and modify curricular activities and/or practices (Kolak et al., 2020) to ensure better alignment between education and societal needs (Ross & Cameron, 2021; Kaprielian et al., 2013). While priority health concerns are to be identified collaboratively alongside key stakeholders (Boelen & Heck, 1995), these indices may be used to establish more impactful collaborations with local health stakeholders (Kolak et al., 2020). Furthermore, schools may elect to use these indices during the admissions process by creating more targeted application components and/or interview questions asking potential applicants about their perceptions of community health needs. Lastly, schools may also decide to use these indices in combination with other internal data to assist in identifying community-based learning opportunities and areas of need (Kolak et al., 2020).

The aim of developing the indices was to provide guidance to advance social accountability in health professions education. The consequential validity of the index lies in its ability to provide insight into the health needs of a respective region. This information may be used by schools to help inform educational practices and perhaps provide the initial steps in being able to generate actionable recommendations to improve population health outcomes. Leveraging open access population health data in a systematic approach serves as a valuable tool for identifying relevant societal needs. This approach could lead to the development of regionally sensitive health profiles, increased agreement of relevant community health needs, more purposeful conversations with community stakeholders, as well as more targeted resource allocation (Kolak et al., 2020). The use of data to support educational improvements has been shown to be effective in improving medical training (Triola et al., 2018; Chahine et al., 2018). Despite calls in the literature to better utilize open access data collected by governments to improve medical training, schools struggle to make these links (Triola et al., 2018; Chahine et al., 2018; Dauphinee, 2012). Few seminal population-based outcome studies have examined the relationship between health professions training and health outcomes (Tamblyn, 2011; Wenghofer et al., 2009; Kawasumi et al., 2011; Cadieux et al., 2007; Norcini et al., 2000; Norcini et al., 2014; Asch et al., 2009; Asch et al., 2014; Epstein et al., 2013, 2016; Teodorczuk et al., 2017). However, this paper provides an example of how schools can begin to utilize open access, secondary data to create a reliable health indices as a means to empirically identify regional population health needs.

Findings of this study utilized open access data to identify priority health needs. Although open access data remains readily available, cost-effective, and generalizable, there are a number of limitations to consider. Despite continual global government invest in the quality and accessibility of publicly available data, the system remains imperfect (Health Canada, 2004). Open access datasets are designed to be representative of the larger population there are often several data control methods and restrictions imposed for confidentiality and anonymity, limiting access to information and variables at smaller levels of geography. Although access to neighbourhood-level data would allow for greater specificity and comparisons across smaller geographical areas, this study identified universal health needs from open access data, accessible to all schools. Further research could include replicating these analyses using restricted data available through affiliated academic research data centres (e.g., Statistics Canada’s Research Data Centres (RDC) or Federal Statistical Research Data Centers (FSRDC) in the United States). Additionally, the speed at which up-to-date data is available is often delayed, which could impact the reliability of the indices over time. While this study utilized the most up-to-date available data at the time of analysis, more timely access to current data should be made more readily available to researchers. These indices should be updated and modified with the release of new CCHS cycle data (approximately every 4 years) to reflect accurate and timely population health needs. This timeline aligns to previous research stating that the half-life of most health professions curricula is 5 years, at which time necessitates the need to examine and revise content (Arthur & Baumann, 1996). However, caution should be used when combining CCHS cycles across years as modules and question response categories often change (Statistics Canada, 2015b).

The indicators included in this study were selected based on their alignment to the CIHF conceptual model. However, the selection of indicators was limited by data availability and may not necessarily reflect a comprehensive list of all possible health indicators. Due to missing data issues, analyses were aggregated to the health region level, reflecting population means, reducing the analytical sample. However, health region level aggregation was deemed appropriate from a theoretical and epistemological perspective. This paper presents a reliable, nationally relevant, regionally sensitive health index measured at administrative regions responsible for administering and delivering health care in Canada. Additionally, there are also several potential other important factors that may be necessary to validate the use of a regional health profile to advance social accountability in medical education, including stakeholder engagement (Boelen & Heck, 1995) (e.g., community members, healthcare providers, and policy makers, etc.), contextual factors (Boelen et al., 2012) (e.g., broader social, economic, and geo-political contexts), longitudinal data (e.g., track changes in health outcomes over time), interprofessional collaboration (Fleet et al., 2008) (e.g., promotion of collaboration among various health professions teams and disciplines), and resource allocation (Global Consensus and for Social Accountability of Medical Schools, 2010) (e.g., financial and human resources). Lastly, the CCHS is representative of self-reported data, and the presence of chronic health conditions cannot be confirmed and may be under/over reported. However, self-reported health metrics are often used as general proxies for health status as they are inexpensive, readily available (Muggah et al. 2013; Miilunpalo et al. 1997; Skinner et al. 2005) and associated with lifestyle-related diseases, lifestyle habits as well as mortality (Li et al., 2020; Yamada et al., 2012; Gallagher et al., 2016; Cislaghi & Cislaghi, 2019).

## Conclusion

The development of a health index is imperative to initiate quality processes to empirically identify societal needs, and serves as a starting point to establish stronger relationships between education and society (Triola et al., 2018). Despite the importance of secondary population health and demographic databases in other fields, health professions education has largely overlooked their use. This study demonstrates how open access, secondary data can be utilized to create reliable health indices that identify population health needs. These indices can be used to align resources, services, and research activities, and inform admissions criteria and curricular design.

Future research should focus on how health professions schools can better utilize secondary data to better inform and understand priority health needs as well as the socio-demographic composition of the populations they serve. This information may be used to better inform health workforce need, admission processes, underservice areas, future health care needs, and curricular design to ensure social determinants of health are integrated throughout the curriculum. Schools must utilize their resources in a more purposeful way and ensure that graduates acquire the competencies most relevant to societal needs (Boelen et al., 2019). Additionally, future work could also focus on how schools can better identify their mandated geographic service areas using preidentified government regions or administrative areas such as health regions, census divisions or subdivisions.

This study provides an example of a systematic and iterative approach to developing a socially accountable health index using pan-national open access secondary data. The indices created in this study serve as a proxy for societal health needs and perhaps may provide a starting point for establishing stronger relationships between education and society. It is important for schools to utilize their resources more purposefully and ensure that graduates are equipped with the competencies needed to address societal needs (Boelen & Woollard, 2009). Closing the gap between education and society has the potential to improve health outcomes (Triola et al., 2018), and promote a more socially accountable health professions education system.

## Appendix

Canadian Provinces and Territories and Affiliated Health Regions included in the Canadian Community Health Survey (CCHS) 2017–18 Public Use Micro-Data File (PUMF) (n = 97).

**Table:**

Province or Territory^a^	Health regions^b^
Newfoundland & Labrador	Eastern Regional
	Central Regional
	Western Regional Health Authority & Labrador-Grenfell Regional Health
Prince Edward Island	Prince Edward Island
Nova Scotia	Zone 1–Western
	Zone 2–Northern
	Zone 3–Eastern
	Zone 4–Central
New Brunswick	Zone 1 (Moncton area)
	Zone 2 (Saint John area)
	Zone 3 (Fredericton area)
	Zone 4 (Edmundston area) & Zone 5 (Campbellton area)
	Zone 6 (Bathurust area) & Zone 7 (Miramichi area)
Quebec	Bas-Saint-Laurent
	Capitale-Nationale
	Chaudière-Appalaches
	Région de Laval
	Région de Lanaudière
	Région des Laurentides
	Saguenay-Lac-Saint-Jean
	L'Estrie
	Gaspésie-Îles-de-la-Madeleine
	Mauricie et du Centre-du-Québec
	Région de Montréal
	Montérégie
	L'Outaouais
	L'Abitibi-Témiscamingue
	Côte-Nord
Ontario	Brant county health unit
	City of hamilton health unit
	Niagara Regional area health unit
	Waterloo health unit
	North Bay parry sound & timiskaming
	Northwestern health unit
	Porcupine health unit
	Renfrew county and district health unit
	Sudbury and district health unit
	The District of algoma health unit
	Thunder Bay District health unit
	City of Ottawa health unit
	Eastern Ontario health unit
	Haliburton, Kawartha, Pine Ridge district health unit
	Leeds, Grenville and Lanark district health unit
	Hastings and Prince Edward counties health unit
	Kingston, Frontenac and Lennox and Addington health unit
	City of Toronto health unit
	Durham Regional health unit
	Halton Regional health unit
	Peel Regional Health Unit
	Peterborough County-City health unit
	Simcoe Muskoka District health unit
	Wellington-Dufferin-Guelph health unit
	York Regional health unit
	Chatham-Kent health unit
	Elgin-St Thomas health unit
	Grey Bruce health unit
	Haldimand-Norfolk health unit
	Huron & Perth
	Lambton health unit
	Middlesex-London health unit
	Oxford County Health Unit
	Windsor-Essex County health unit
Manitoba	Winnipeg RHA
	Prairie Mountain health
	Interlake-Eastern regional health
	Northern Regional health area
	Southern health
Saskatchewan	Sun Country Regional health authority & Five Hills regional health authority & Cypress regional health authority
	Regina Qu’Appelle RHA
	Sunrise Regional health authority & Kelsey Trail Regional health authority
	Saskatoon RHA
	Heartland Regional Health Authority & Prairie North regional health authority
	Prince Albert Parkland regional health authority
Alberta	South zone
	Calgary zone
	Central zone
	Edmonton zone
	North Zone
British Columbia	East Kootenay HSDA
	Kootenay-Boundary HSDA
	Okanagan HSDA
	Thompson/Cariboo HSDA
	Fraser east HSDA
	Fraser north HSDA
	Fraser south HSDA
	Richmond HSDA
	Vancouver HSDA
	North Shore/Coast Garibaldi HSDA
	South Vancouver Island HSDA
	Central Vancouver Island HSDA
	North Vancouver Island HSDA
	Northwest HSDA
	Northern interior HSDA
	Northeast HSDA
Yukon	Yukon
Northwest Territories	Northwest Territories
Nunavut	Nunavut

^a^There are 10 provinces and 3 Territories in Canada

^b^Health Regions refer to administrative areas defined by the provincial ministries of health and are used to make health care decisions
